# Offspring of women with hyperemesis gravidarum are more likely to have cardiovascular abnormalities

**DOI:** 10.1186/s12884-024-06293-6

**Published:** 2024-02-08

**Authors:** Jiao Fan, Minghong Yin

**Affiliations:** Shanghai Key Laboratory of Maternal Fetal Medicine, Shanghai Institute of Maternal-Fetal Medicine and Gynecologic Oncology, Shanghai First Maternity and Infant Hospital, School of Medicine, Tongji University, Shanghai, China

**Keywords:** Hyperemesis gravidarum, Cardiovascular abnormalities, Ketone body

## Abstract

**Background:**

Hyperemesis gravidarum (HG) is a severe form of pregnancy-related nausea and vomiting affecting 0.3–2.3% of pregnancies, which can lead to fluid, electrolyte, and acid–base imbalances, nutritional deficiencies, and weight loss, and is usually severe enough to require hospitalization. Abnormally elevated urinary ketones are commonly seen in patients with HG, and ketone bodies are free to pass through the placenta, and maternal hyperketonemia, with or without acidosis, is associated with an increased rate of stillbirth, an increased incidence of congenital anomalies, and impaired neurophysiologic development of the infant. This study investigates the obstetric outcomes of patients with HG and whether HG increases the incidence of cardiovascular disease in the offspring.

**Methods:**

This study included 1020 pregnant women who were hospitalized in our hospital for HG and ultimately delivered in our hospital as well as pregnant women without HG in early gestation and delivered in our hospital from January 2019-January 2020, and we collected and followed up the clinical information of the pregnant women and their offspring.

**Results:**

Pregnant women with HG were more likely to have severe urinary ketones, the rate of early miscarriage and mid-term miscarriage was significantly higher in women with HG compared to pregnant women without HG. Fetal and neonatal head and abdominal circumferences were smaller in HG group than in control group.

Neonatal birth weight and length were also lower in the HG group and cardiovascular anomalies were more likely to occur in the offspring of women with HG when all births were followed up for 3 years.

**Conclusions:**

HG may cause poor obstetric outcomes and was associated with the development of cardiovascular disease in the offspring of women with HG.

## Introduction

Many women suffered from nausea and vomiting in early pregnancy: it has been estimated that the incidence was as high as 70% of pregnancies [1]. HG (HG) is a severe form of nausea and vomiting during pregnancy. HG is associated with fluid, electrolyte, and acid–base imbalances, nutritional deficiencies, and weight loss, and is often severe enough to require hospitalization. Generally, about 60% of HG cases resolved by the end of early pregnancy, and up to 90% of cases resolved by 20 weeks of gestation [2]. Studies have concluded that ketonuria and high serum urea were particularly valid laboratory indicators of the severity of HG, as they were good markers of starvation and dehydration [3]. Ketosis implies heavy use of fat stores and fat metabolism. Similarly, urea is a byproduct of protein metabolism, and high serum urea in patients with HG may be due to a combination of protein-energy catabolism metabolism and may be exacerbated by dehydration-induced reduced renal clearance [3]. Although ketonuria was not found to be a marker of HG in a meta-analysis, 65% of the articles included in the meta-analysis were found to have ketonuria in patients with HG only, suggesting that elevated ketone bodies possess some influences in HG [4].

Ketone bodies are ketones that can travel from the mother's bloodstream through the placenta to the fetus [5]. There was substantial evidence that the in utero environment has a lasting impact on the later health of the fetus, and that malnourished mothers, especially in early pregnancy, were associated with an increased risk of chronic degenerative diseases (type 2 diabetes mellitus, cardiovascular disease, and breast cancer) [6–8]. In an analysis of a multiethnic community-based prospective cohort, the study demonstrated that elevated ketone bodies were associated with a range of cardiovascular pathologies and outcomes, including all-cause mortality, cardiovascular disease-related deaths, and heart failure events [9]. HG was connected with various unfavorable perinatal outcomes including low birth weight and premature birth. Additionally, it was discovered that macrosomia and stillbirth were less common complications in pregnancies accompanied by HG [10, 11]. However, other studies have not found an association between HG and low birth weight, and women with HG have a higher incidence of stillbirths [12, 13]. According to a comprehensive study, there may be a slight correlation between HG and an increased risk of testicular cancer, mental health issues, and neurodevelopmental abnormalities [14]. Based on the results of the above literature suggesting that HG could affect fetal development and the adverse cardiovascular effects of ketone bodies, we hypothesized that elevated ketone bodies in women with HG passed through the placenta to the fetus and thereby adversely affected the fetus as well as abnormal cardiovascular development. Therefore, our study investigated whether HG was associated with poor fetal development and the occurrence of cardiovascular disease in the offspring.

## Methods

### Patients

This study included pregnant women who were hospitalized for HG in early pregnancy at our hospital and eventually delivered at our hospital and pregnant women without HG in early pregnancy who delivered at our hospital from January 2019-January 2020, and this study included 510 pregnant women who were hospitalized for HG of pregnancy at 6–10 weeks of gestation as well as 510 pregnant women without HG of pregnancy at 6–10 weeks of gestation as a control, and tracked the outcomes of the pregnancy, the obstetrical outcomes, and the follow up of all the enrolled women. In this retrospective study, we first selected women with HG according to the inclusion criteria, and then matched them according to age, BMI, and parity to select women without HG as a control group. Our cases of HG were characterized as "intractable nausea and vomiting of pregnancy with dehydration and starvation, clinically judged to require hospitalization for intravenous rehydration and antiemetic medication". All pregnant women were excluded if they had twin pregnancies, thyroid disorders, pre-eclampsia, renal disorders, gestational diabetes mellitus, or gastrointestinal disorders during pregnancy. Basic clinical information, gestational conditions, and neonatal births were collected from all pregnant women, and all fetuses born were followed up by telephone 3 years later to inquire about the occurrence of cardiovascular diseases. The pregnancy status included: early miscarriage rate: fetal arrest detected before 14 weeks of gestation, mid-term miscarriage rate: fetal arrest detected between 14 and 27 weeks of gestation, collection of ultrasound examination between 22 and 24 weeks of gestation to measure fetal head circumference, abdominal circumference, whether the four chambers of the heart were visible or not, and the rate of preterm and full-term labor. The birth of newborns, including newborns' birth weight, length, head circumference, abdominal circumference, appearance of deformities, and cardiac auscultation, and the follow up of all the births after 3 years: cardiovascular diseases occurrence. The study was approved by the Ethics Committee of Shanghai First Maternity and Infant Hospital, and all patients signed an informed consent form.

### Sample size calculation

The primary outcome of this study was the incidence of cardiovascular diseases. The comparison of the incidence rates of the independent samples was the offspring cardiovascular incidence: 10% incidence predicted in the group of HG and 1% in the group of no HG. The incidence percentages were chosen according to data from our hospital's medical records. The difference between the two groups with respect to the clinical significance was 5%, α:0.05, degree of certainty(1 -β):0.8,and the ratio of the sample size of the two groups was 1:1.The sample size required for each group was 504.In this study 510 patients were included in each group.

### Statistical analysis

SPSS 20 software (SPSS Inc., Chicago, USA) was used for statistical analysis, and quantitative data were expressed as mean ± standard deviation. Comparisons of quantitative data between two groups were performed using the t-test or Wilcoxon rank-sum test, and comparisons of qualitative data were performed using the chi-square test or Fisher exact test. Rank variables were compared by Kruskal Wallis rank sum test. p-values less than 0.05 were statistically different.

## Results

Significantly elevated ketone bodies in women with HG.

There was no difference in the basic clinical data of age, BMI, parity, and gestational week between women in the HG group and women in the no HG group, but we found that urinary ketone levels were significantly higher in women in HG group than in women in the no HG group, and the probability of severe urinary ketones in the women in HG group was significantly higher (69.1%) than that in the women in the no HG group (1.1%) (Table 1).

**Table 1:** Basic clinical data on women in the group with and without HG during early pregnancy

Variables	HG (*n* = 510)	No HG (*n* = 510)	*P* value
Age(y)	29.50 ± 4.54	29.95 ± 4.89	0.12
BMI	20.61 ± 1.22	20.74 ± 1.26	0.08
Gravidity	2.42 ± 0.61	2.40 ± 0.66	0.58
Parity	0.96 ± 0.32	0.93 ± 0.38	0.25
Gestational age at admission(d)	58.56 ± 4.85	59.10 ± 5.01	0.07
Ketonuria			< 0.0001
-	7 (1.3%)	472 (92.5%)	
1 +	24 (4.7%)	23 (4.5%)	
2 +	127 (24.9%)	10 (1.9%)	
3 +	352 (69.1%)	5 (1.1%)	
Ketonuria			< 0.0001
Mild(1 + to 2 +)	151 (29.6%)	33 (6.4%)	
Severe (3 +)	352 (69.1%)	5 (1.1%)	

Data were shown as mean ± SD or % (n/N). t-test and chi-squared analysis were used. *p*-value of less than 0.05 was considered to be statistically different

### Women with HG were more likely to have a miscarriage during pregnancy

The rate of early and mid-term miscarriage was significantly higher in the women with HG than in the women without HG, and the ultrasound results of the patients at 22–24 weeks of gestation suggested that the head and abdominal circumference of the fetuses in the women with HG group was significantly smaller than that of the fetuses in the women without HG, and that the percentage of fetuses with the four chambers of the heart invisible in ultrasound was 5.8% in women with HG, whereas the percentage of fetuses with the four chambers of the heart invisible in ultrasound was only 1.7% in women in the no HG group. However, our data did not reveal a higher rate of preterm birth in the women with HG than in the women without HG (Table 2).

**Table 2:** Pregnancy data on women in the group with and without HG

Variables	HG	No HG	*P* value
First trimester abortion rate	7.4% (38/510)	3.5% (18/510)	0.008
Mid-term abortion rate	4.0% (19/472)	1.6% (8/492)	0.031
Head circumference(cm)	19.88 ± 0.74	21.50 ± 0.51	< 0.0001
Abdomen circumference(cm)	18.19 ± 0.83	19.04 ± 0.68	< 0.0001
Four-chamber view of fetal hearts			0.001
Visible	94.2% (427/453)	98.3% (476/484)	
No visible	5.8% (26/453)	1.7% (8/484)	
preterm birth	9.1% (39/427)	6.9% (33/476)	0.268

Data were shown as mean ± SD or % (n/N). t-test and chi-squared analysis were used. *p*-value of less than 0.05 was considered to be statistically different

### Neonatal development was worse in women with HG than in the group of no HG

The birth weight, length, head circumference, and abdominal circumference of newborns were smaller in the women with HG than in the women without HG, and there were no obvious malformations in the newborns of either group; however, there were significantly more newborns in the women with HG who had audible murmurs on cardiac auscultation than in the group of women without HG (Table 3).

**Table 3:** Data of newborns in the group with and without HG

Variables	HG	No HG	*P* value
Birth weight(kg)	2.99 ± 0.35	3.90 ± 0.23	< 0.0001
Birth length(cm)	48.98 ± 0.99	50.44 ± 0.71	< 0.0001
Head circumference(cm)	33.29 ± 0.97	33.96 ± 0.35	< 0.0001
Abdomen circumference(cm)	33.63 ± 1.12	35.67 ± 0.48	< 0.0001
Appearance deformity	0	0	-
Cardiac auscultation			< 0.0001
No noise heard	89.5% (382/427)	97.4% (464/476)	
Noise heard	10.5% (45/427)	2.6% (12/476)	

Data were shown as mean ± SD or % (n/N). t-test and chi-squared analysis were used. *p*-value of less than 0.05 was considered to be statistically different

### Offspring of women with HG were more likely to develop cardiovascular diseases

Telephone follow-up of cardiovascular diseases in the offspring of women in both groups 3 years after birth revealed that the offspring of women in the HG group were more likely to have cardiac developmental abnormalities and cardiac macrovascular abnormalities than the offspring of women in the no HG group (Table 4), cardiac developmental abnormalities including atrial and ventricular structural abnormalities, ventricular septal and atrial septal defects, etc. Abnormalities of cardiac macrovascular, including the mitral valve, tricuspid valve, aortic valve, and pulmonic valve, were seen in abnormal blood flow.

**Table 4:** Cardiovascular disease in fetuses 3 years after birth in the HG and No HG groups

Variables	HG (*n* = 427)	No HG (*n* = 476)	*P* value
Cardiac dysplasia	31 (7.2%)	6 (1.3%)	< 0.0001
Cardiac vascular abnormality	29 (6.7%)	5 (1.1%)	< 0.0001

Data were shown as % (n/N). chi-squared analysis was used

*p*-value of less than 0.05 was considered to be statistically different

## Discussion

In our study, we found that ketone body levels were significantly elevated in women with HG relative to women without HG, the proportion of fetuses with four chambers of the heart not visible on mid-pregnancy ultrasound was significantly higher in women with HG than in women without HG, the proportion of newborns with an audible cardiac murmur at birth was significantly higher in women with HG than in women without HG. The long-term outcome of the offspring of women with HG was also found to be more susceptible to cardiovascular abnormalities, so we believe that HG may have adverse short- and long-term effects on the offspring of women.

The strengths of our study are that we included a total of 1020 women and we collected their outcomes from early pregnancy to three years postpartum in their offspring and we can determine that HG has a detrimental cardiovascular effect on the offspring, our limitation is that this is a retrospective study, and there may be a selection bias, but our use of matched control patients reduces the selection bias, and also we extracted the same way for the two groups of patients and there was no missing data, so information bias was avoided.

HG were defined according to Fairweather's criteria [15], i.e., vomiting that occurred in the first 20 weeks of pregnancy and was severe enough to require hospitalization, but was not associated with episodic illnesses such as appendicitis or pyelitis. Normal pregnancy results in increased metabolic rate and mild ketosis [15, 16]. Early in pregnancy, nausea and vomiting will exacerbate ketosis in women, with a rapid rise in ketones at 20 h of fasting. The metabolism of women with HG is similar to that of fasting and starving women, producing hyperketonemia [17].

Research from the Dutch famine suggested that offspring conceived during the famine had an increased risk of developing diabetes later in life [6]. Offspring of HG women were found to have lower insulin sensitivity in childhood [18]. The majority of women with HG required recurrent medical care, 60% of pregnant women with HG were readmitted to the hospital, and ≥ 5 births, multiple births, and female sex of the fetus were associated with higher odds of readmission, maternal age 36–40 years, BMI ≥ 35 kg/ m2, smoking, and assisted reproductive technology were associated with lower odds of readmission [19]. A meta-analytic study [20] found that women with HG during pregnancy were more likely to have girls and that female fetal sex was significantly associated with high serum urea and severe ketonuria. They were also more likely to have a lower birth weight baby, and had a higher incidence of both small-for-gestational-age and preterm birth. Similarly, other studies have also shown that women with HG in early pregnancy were more likely to have low birth weight babies [10, 11]. These findings implied that the metabolic disturbances produced during vomiting may lead to an unfavorable intrauterine environment, which may affect infant growth. Therefore, women with HG should be considered as a high-risk group and closely monitored for intrauterine growth retardation and malformations in the later stages of pregnancy [21]. However, in other study the fetuses of women with HG have not been found to be of low birth weight [13], and a detailed look at the article revealed that the number of cases included in this study was small and that the thyroid hormone levels in women with HG were significantly higher than those in the control group, so it may be that thyroid dysfunction also has an impact on the birth weight of the fetuses [13]. The results of studies on the relationship between HG and stillbirth were also controversial, with study reporting that stillbirth was more common in patients with HG [12]. Nevertheless, in a population-based cohort study, a significant decrease in the incidence of stillbirths was found in women with HG [11]. In short, current studies on the association of HG with pregnancy outcomes and perinatal outcomes was inconclusive and may be related to the population included in studies, the number of cases, and the methodology used in the study. Our results showed that although the mid-gestational head circumference, abdominal circumference, and weight, length, head circumference, and abdominal circumference of newborns were significantly lower in women with HG than in women without HG, we did not find that preterm labor was more likely to occur in women with HG.

Pregnancy is related to a 2–threefold increase in maternal ketone body levels from baseline levels, and these levels increase rapidly and exaggeratedly in fasting [22]. Ketone bodies are free to cross the placenta. In rodent models, prolonged high maternal ketone body levels have been associated with fetal malformation formation, including neural tube defects [23]. It has been known for many years that extension of the maternal ketone body state, whether physiologic or pathologic, can adversely affect fetal brain development [24]. A study [25] found that fasting and late-gestational maternal ketone body levels in 188 women with diabetes or gestational diabetes were associated with impaired mental development in the offspring, after correcting for socioeconomic status, race, and ethnic origin. Ketone bodies are an important alternative metabolic fuel source for the myocardium. Studies have shown that elevated ketone bodies levels were associated with disease severity and poor prognosis in patients with heart failure and arrhythmogenic right ventricular cardiomyopathy [26, 27]. In healthy community populations, elevated endogenous ketone bodies were associated with increased cardiovascular disease and mortality [9]. Ketone bodies may serve as potential biomarkers for cardiovascular risk assessment [9]. Although there were ample evidences from human and animal studies that malnutrition during pregnancy increased the risk of chronic diseases [28, 29], there was a lack of studies related to other long-term health effects, such as fetal cardiovascular disease, as a result of HG. First indications from a small prospective cohort research indicated that women with more severe HG have more ApoB, a marker of cardiometabolic disease, in umbilical vessels in offspring at birth [30]. A study suggested that elevated levels of 3-hydroxybutyric acid in late pregnancy can adversely affect a child's intelligence [25]. To our knowledge, there was only one report of an increased risk of testicular cancer in boys born to mothers with HG, presumably due to elevated estrogen levels during testicular differentiation [14]. It is because ketone bodies could have adverse cardiovascular effects, abnormally elevated ketone bodies in women with HG can pass through the placenta into the fetus, high ketone body levels may affect fetal cardiovascular development, and there is a lack of studies on the long term outcomes of the offspring of women with HG, we followed the outcomes of the offspring of women with HG for three years and found that the offspring of women with HG had significantly higher cardiovascular morbidity than the offspring of women without HG.

## Conclusion

Although maternal urinary ketones can be relieved by glucose supplementation, short-term nutritional deficiencies in pregnant women with HG will result in abnormally high ketone body levels that rapidly enter the placenta and reach the fetus, with consequent effects on the fetus. This article found that HG was associated to poor obstetric outcomes and long-term adverse effects on the fetus: the incidence of cardiovascular disease was significantly increased, which might be related to elevated ketones. Certainly, the role of ketone bodies in HG needs to be proved by further research.

## Data Availability

No datasets were generated or analysed during the current study.
